# One-plasmid double-expression His-tag system for rapid production and easy purification of MS2 phage-like particles

**DOI:** 10.1038/s41598-017-17951-5

**Published:** 2017-12-13

**Authors:** Pavel Mikel, Petra Vasickova, Petr Kralik

**Affiliations:** 1 Veterinary Research Institute, Hudcova 296/70, 621 00 Brno, Czech Republic; 2 Department of Experimental Biology, Faculty of Science, Masaryk University, Kamenice 753/5, 625 00 Brno, Czech Republic

**Keywords:** Bacteriophages, Bacteriophages, Phage biology, Phage biology

## Abstract

MS2 phage-like particles (MS2 PLP) are artificially constructed pseudo-viral particles derived from bacteriophage MS2. They are able to carry a specific single stranded RNA (ssRNA) sequence of choice inside their capsid, thus protecting it against the effects of ubiquitous nucleases. Such particles are able to mimic ssRNA viruses and, thus, may serve as the process control for molecular detection and quantification of such agents in several kinds of matrices, vaccines and vaccine candidates, drug delivery systems, and systems for the display of immunologically active peptides or nanomachines. Currently, there are several different *in vivo* plasmid-driven packaging systems for production of MS2 PLP. In order to combine all the advantages of the available systems and to upgrade and simplify the production and purification of MS2 PLP, a one-plasmid double-expression His-tag system was designed. The described system utilizes a unique fusion insertional mutation enabling purification of particles using His-tag affinity. Using this new production system, highly pure MS2 PLP can be quickly produced and purified by a fast performance liquid chromatography (FPLC) approach. The system can be easily adapted to produce other MS2 PLP with different properties.

## Introduction

MS2 phage-like particles (MS2 PLP) are artificial pseudo-viral particles whose production is based on knowledge of the familiar bacteriophage MS2 (+ssRNA, *Leviviridae*)^1^ and the ability of its coat protein dimers to pack specific single-stranded RNA (ssRNA) molecules^2–4^. The packaging is driven by dimerization of coat proteins followed by a specific interaction of dimers with a stem-loop structure (called the “*pac*” site) in ssRNA resulting in spontaneous formation of MS2 PLP. These particles are small in size (about 27 nm), non-enveloped, non-infectious, non-replicative, stable pseudo-viral particles with the ability to protect encapsidated ssRNA molecules against the effects of ubiquitous nucleases. Due to these properties and their morphological and physicochemical characteristics, MS2 PLP carrying specific ssRNA molecules can serve as suitable process control virus (PCV) for the detection and quantification of ssRNA viruses within a wide range of samples; for example, human norovirus (NoV), human enterovirus (EV), hepatitis A virus (HAV), hepatitis E virus (HEV), hepatitis C virus (HCV), and human immunodeficiency virus type 1 (HIV-1)^2,5–8^. Perspectives on the practical use of MS2 PLP as RNA vaccines and vaccine candidates, drug delivery systems, and systems for the display of immunologically active peptides or nanomachines are discussed in a recent review^9^.

Since the first study at the beginning of the 1990s in which non-bacteriophage RNA was encapsulated by MS2 coat protein dimers, many different methods for the production of MS2 PLP have been developed^4^. Currently, the production of MS2 PLP is based on various *in vivo* plasmid-driven packaging systems, which exploit the spontaneous assembly of MS2 coat protein dimers in the presence of the *pac* site inside *Escherichia coli* (*E*. *coli*)^10^. However, while each of these systems have their advantages, they are also characterized by various drawbacks, e.g., limited capacity for packaging of control ssRNA, lower efficiency of packaging, complicated system construction, or complicated and time-consuming purification of produced MS2 PLP. The newest and most advanced plasmid-driven packaging system, the so-called one-plasmid double-expression packaging system, allows construction of MS2 PLP with high efficiency and packaging specificity, and allows a maximum length of encapsidated ssRNA of up to 3,600 nucleotides (nt)^11^. Recently, this packaging system was even used for the construction of MS2 PLP carrying a 4,942-nt-long control sequence for Zika virus^12^. However, this system still does not address a major drawback of current MS2 PLP preparations: the purification procedure. Therefore, produced MS2 PLP must be purified in a time-consuming and laborious ultracentrifugation step, which does not isolate MS2 PLP in the required purity^4,8,10,11,13^. The problem of purification can be solved by the introduction of an affinity tag (e.g., polyhistidine-tag; His-tag) to the structure of MS2 PLP allowing their purification using the Co^2+^ affinity chromatography method^13,14^. This approach enables easy and fast purification of huge amounts of pure MS2 PLP. As the one-plasmid double-expression packaging system had not yet been developed at that time, Cheng *et al*. used a so-called one-plasmid expression system for production of MS2 PLP. The system has limited capacity for packaging of control sequences (up to 2,000 nt), and the particles contain undesirable sequences from bacteriophage MS2^10,13^. Based on this knowledge, none of the previously used plasmid-driven systems for production of MS2 PLP is ideal. The novelty of the present study lies not only in combination of all the experience acquired so far with the different methods of MS2 PLP construction for developing a new plasmid-driven construction system, which integrates all the advantages and benefits of the previously described systems. This is also the first report about the construction of single-chain version of the coat protein dimer containing the His-tag capable to successfully form intact His-tagged MS2 PLP that can be purified in less than one hour by FPLC. Moreover, presented approach solves the previous problem of introduction of His-tag to structure of the coat protein in parallel with formation of whole intact MS2 PLP.

## Results

### Construction of pACYCDuet-1-TM

**Figure 1:**
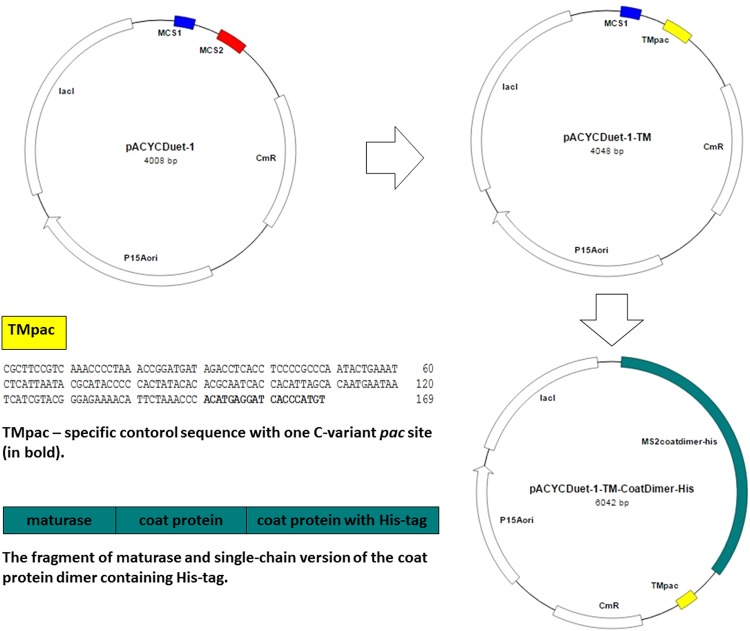
Diagram of cloning experiments in this study. A specific control sequence derived from the mitochondrial DNA (mtDNA) sequences of two extinct species with one C-variant *pac*-site (TMpac) was cloned into the multiple cloning site 2 (MCS2) of the pACYCDuet-1 vector (Novagen, Merck, Germany) to construct the pACYCDuet-1-TM vector. Then, the fragment of maturase and single-chain version of the coat protein dimer containing the His-tag was cloned into the multiple cloning site 1 (MCS1) of the pACYCDuet-1-TM to create the pACYCDuet-1-TM-CoatDimer-His vector.

A specific control sequence derived from the mitochondrial DNA (mtDNA) sequences of two extinct species was cloned into the MCS2 of pACYCDuet-1 to construct the pACYCDuet-1-TM vector (Fig. 1). The resulting construct was verified by sequencing (data not shown).

### Construction of pACYCDuet-1-TM-CoatDimer-His

**Figure 2:**
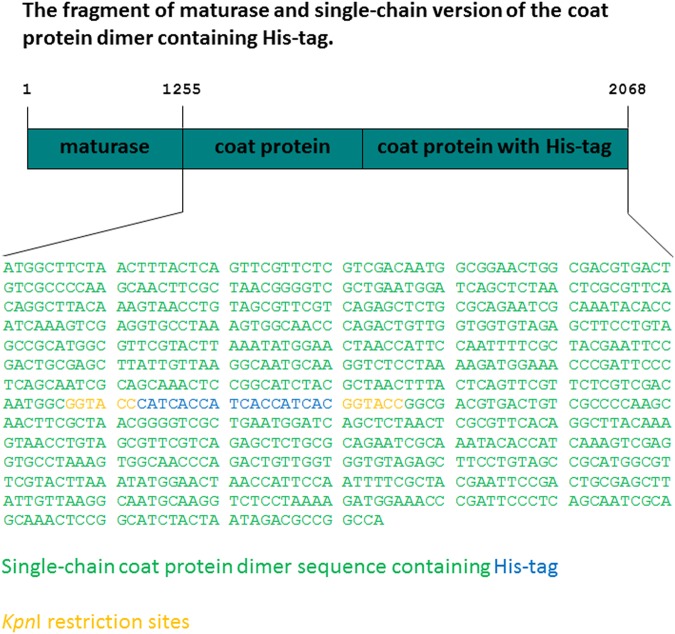
Schematic representation of *de novo*-synthesized sequence of maturase and single-chain version of the coat protein dimer containing the His-tag. *De novo*-synthesized sequence was used as a PCR template from which the fragment of maturase and single-chain version of the coat protein dimer containing the His-tag was amplified and subsequently cloned to multiple cloning site 1 (MCS1) of the pACYCDuet-1-TM. The sequence in green is the sequence of single-chain coat protein dimer containing His-tag (in blue) surrounded by two *Kpn*I restriction sites (in orange) which is localized between nucleotides 1255–2068 in *de novo*-synthesized sequence. Full length *de novo*-synthesized sequence is presented in Supplementary Figure 1.

The fragment of maturase and single-chain version of the coat protein dimer containing the His-tag was amplified from *de novo*-synthesized sequence (Fig. 2), and cloned into the MCS1 of pACYCDuet-1-TM to create the pACYCDuet-1-TM-CoatDimer-His vector. The construct was verified by sequencing (data not shown). DNA fragments were reliably amplified without changes in nt sequence by a DNA polymerase with proofreading activity.

### Induction of expression of His-tagged MS2 PLP

The pACYCDuet-1-TM-CoatDimer-His plasmid was transformed into *E*. *coli* LB21 (DE3) cells and the production of His-tagged MS2 PLP was induced as described below.

### Purification of His-tagged MS2 PLP

**Figure 3:**
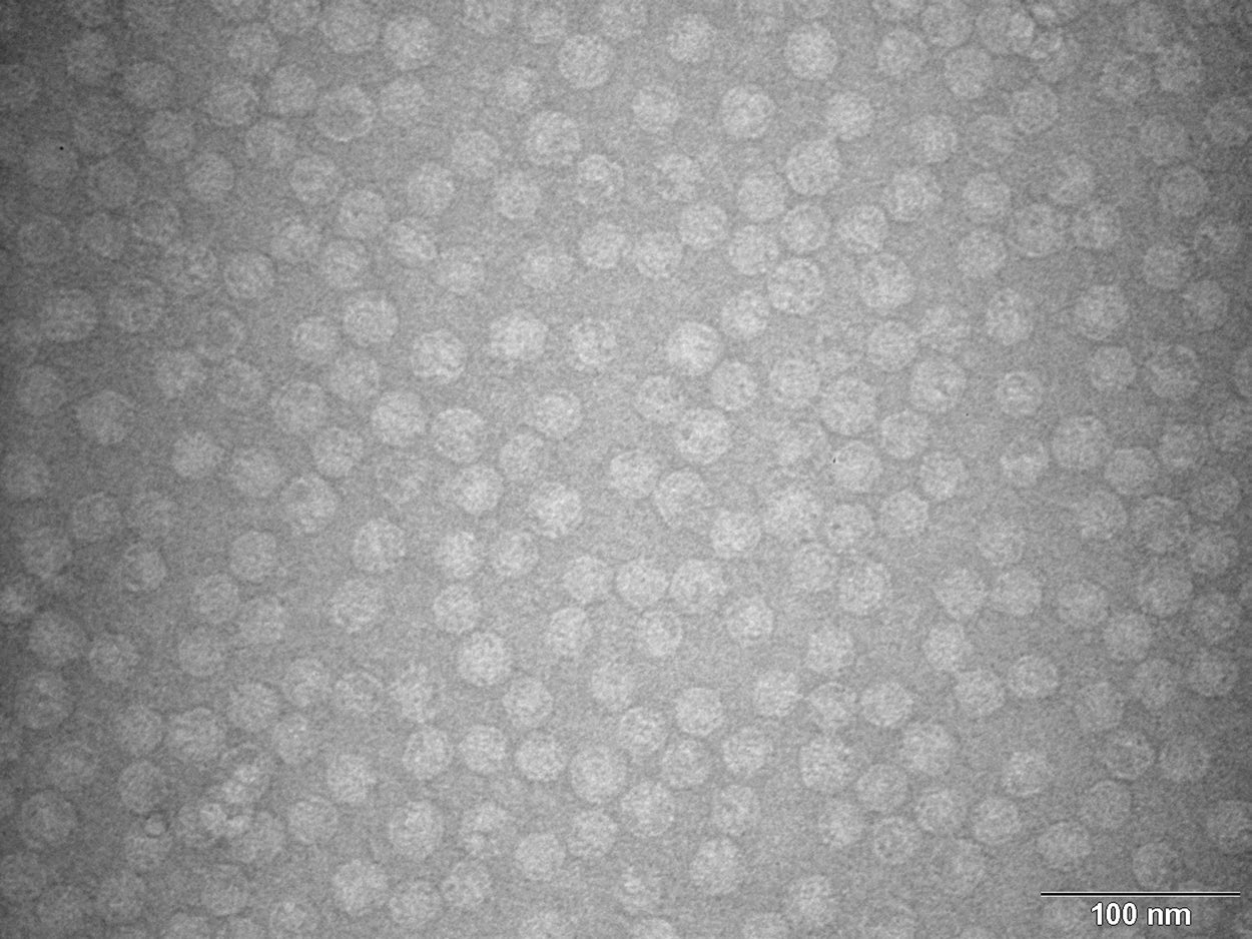
Transmission electron microscopy (TEM) photograph of purified His-tagged MS2 phage-like particles (His-tagged MS2 PLP). The scale is 100 nm.

His-tagged MS2 PLP were purified as described below using an FPLC approach. The integrity of His-tagged MS2 PLP isolated using FPLC was verified by TEM (Fig. 3). The TEM images clearly show that the His-tagged MS2 PLP were correctly assembled into intact capsids of approximately 27 nm in diameter. The His-tagged MS2 PLP were not damaged by the purification process, were isolated in a large quantity (2 × 10^10^ particles/µl; RT-qPCR quantification according to^8^), and did not form any aggregates (Fig. 3).

### Characterization of the single-chain version of the coat protein dimer containing the His-tag

**Figure 4:**
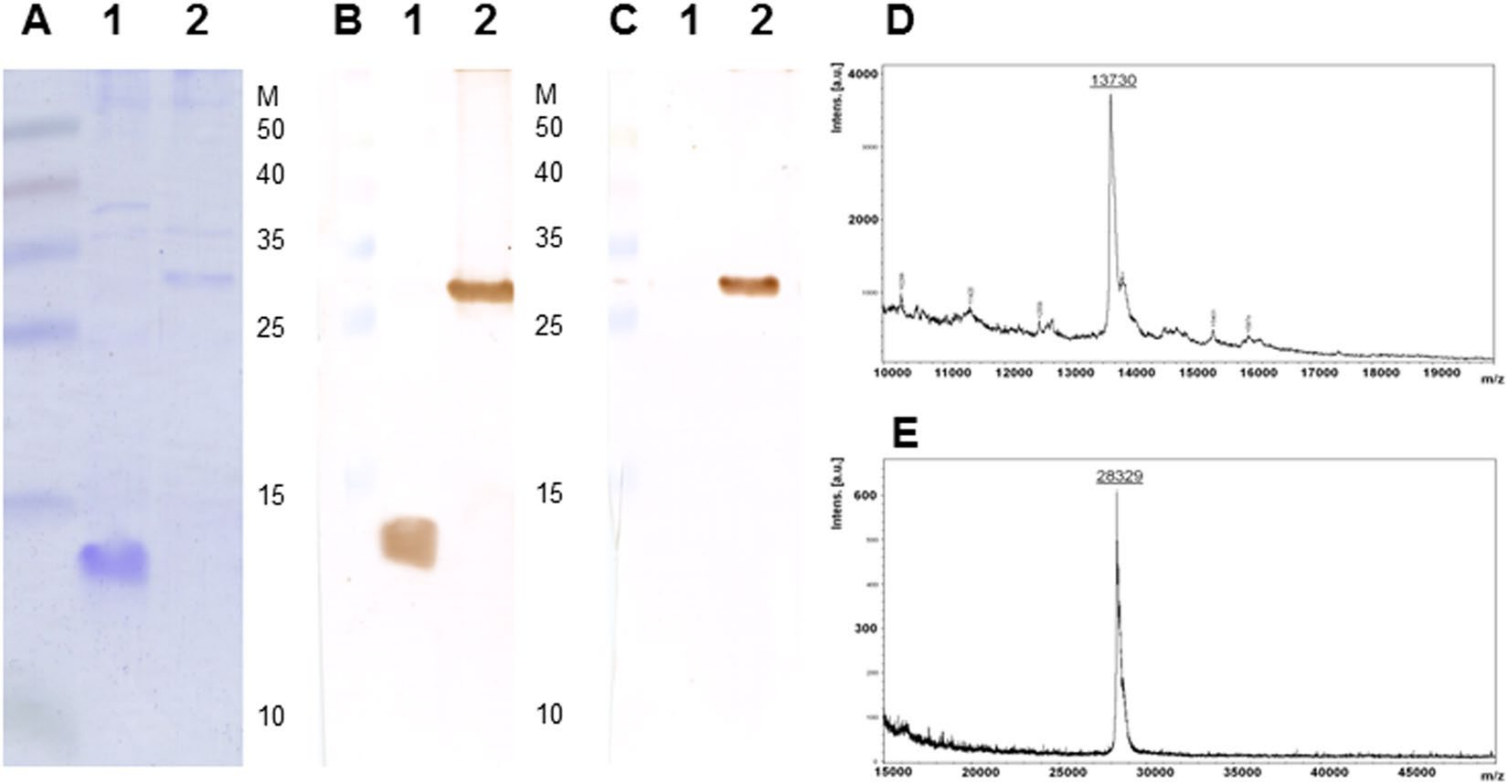
Characterization of the single-chain version of the coat protein dimer containing the His-tag. (**A**) Sodium dodecyl sulfate-polyacrylamide gel electrophoresis (SDS-PAGE), molecular weight of wild type MS2 bacteriophage coat protein was about 13 kDa (A1) whereas single chain version of the MS2 coat protein dimer containing the His-tag was about 28 kDa (A2); (**B**) western blot analysis using primary Anti-Enterobacterio Phage MS2 Coat Protein polyclonal antibody (Merck Millipore, USA) and secondary goat anti-rabbit IgG conjugated with HRP (Jackson ImmunoResearch, UK) antibodies at wild type MS2 bacteriophage coat protein (B1) and single chain version of the MS2 coat protein dimer containing the His-tag (B2); (**C**) western blot analysis using primary anti-HisTag antibody (Pierce, Thermo Scientific, USA) and secondary goat anti-mouse IgG conjugated with HRP (Jackson ImmunoResearch) antibodies at wild type MS2 bacteriophage coat protein (C1) and single chain version of the MS2 coat protein dimer containing the His-tag (C2); (**D**,**E**) laser desorption ionization-time of flight mass spectrometry (MALDI-TOF) analysis of wild-type bacteriophage MS2 coat protein and single-chain version of the MS2 coat protein dimer containing the His-tag; M, marker, spectra multicolor broad range protein ladder (Fermentas); the marker values are in kDa. The figures were cropped, full length gel and blots are presented in Supplementary Figures 2, 3 and 4.

The results of sodium dodecyl sulfate-polyacrylamide gel electrophoresis (SDS-PAGE) showed that MS2 PLP contained wild-type coat protein with an approximate molecular weight of 13 kDa (Fig. 4,A1), whereas His-tagged MS2 PLP incorporated a single-chain version of the coat protein dimer containing a His-tag with an approximate weight of 29 kDa (Fig. 4,A2). While western blot analysis using the Anti-Enterobacterio Phage MS2 Coat Protein polyclonal antibody identified both wild-type (Fig. 4,B1) and single-chain versions of the coat protein dimer containing the His-tag (Fig. 4,B2), the anti-HisTag antibody specifically interacted only with the single-chain version of the coat protein dimer containing the His-tag (Fig. 4,C2). To calculate the exact molecular weight of wild-type and single-chain versions of the coat protein dimer containing the His-tag, matrix assisted laser desorption ionization-time of flight mass spectrometry (MALDI-TOF) analysis was performed. The measured molecular weight of the wild-type coat protein was 13,730 Da (Fig. 4D). The measured molecular weight of the single-chain version of the coat protein dimer containing the His-tag was 28,329 Da (Fig. 4E).

### Stability of His-tagged MS2 PLP against nucleases

**Figure 5:**
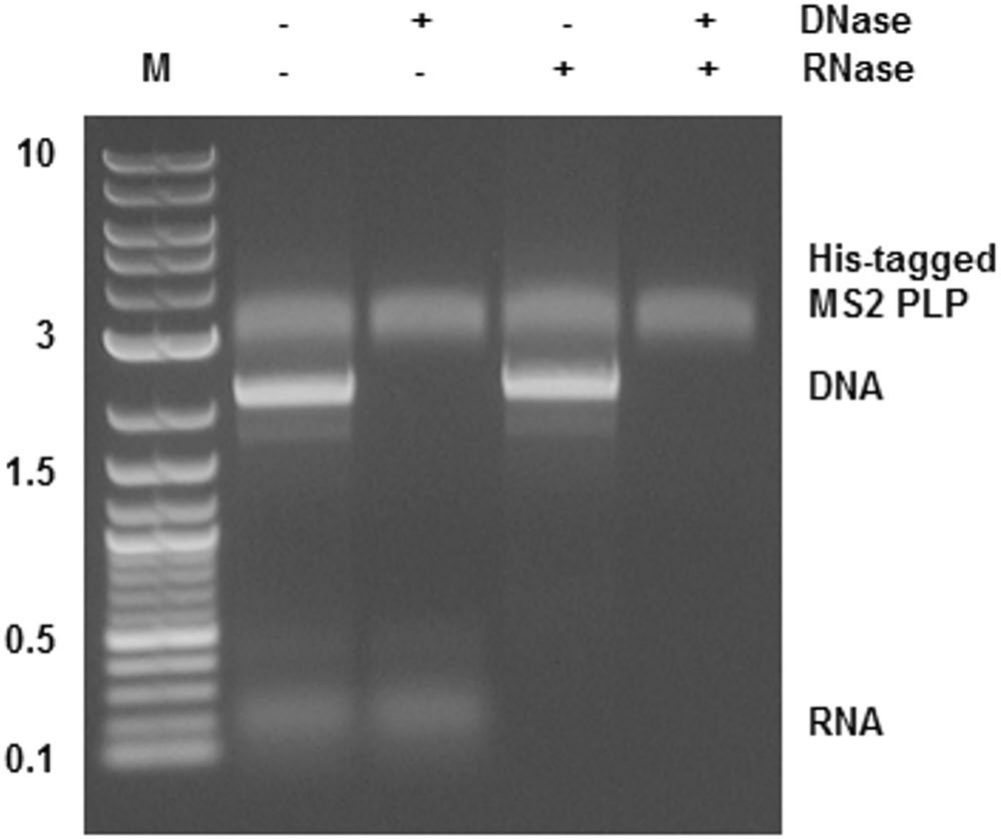
His-tagged MS2 phage-like (His-tagged MS2 PLP) particles stability testing. Agarose gel electrophoresis of His-tagged MS2 PLP, DNA and RNA treated (+) and not treated (−) with DNase and/or RNase; M, marker, 2-log DNA ladder (New England Biolabs, UK); the marker values are in kb. The figure was cropped, full-length gel is presented in Supplementary Figure 5.

Testing of the stability of His-tagged MS2 PLP against nucleases showed them to be resistant under conditions where naked DNA and/or RNA were rapidly degraded (Fig. 5).

### Comparison of temperature stability of MS2 PLP and His-tagged MS2 PLP

**Figure 6:**
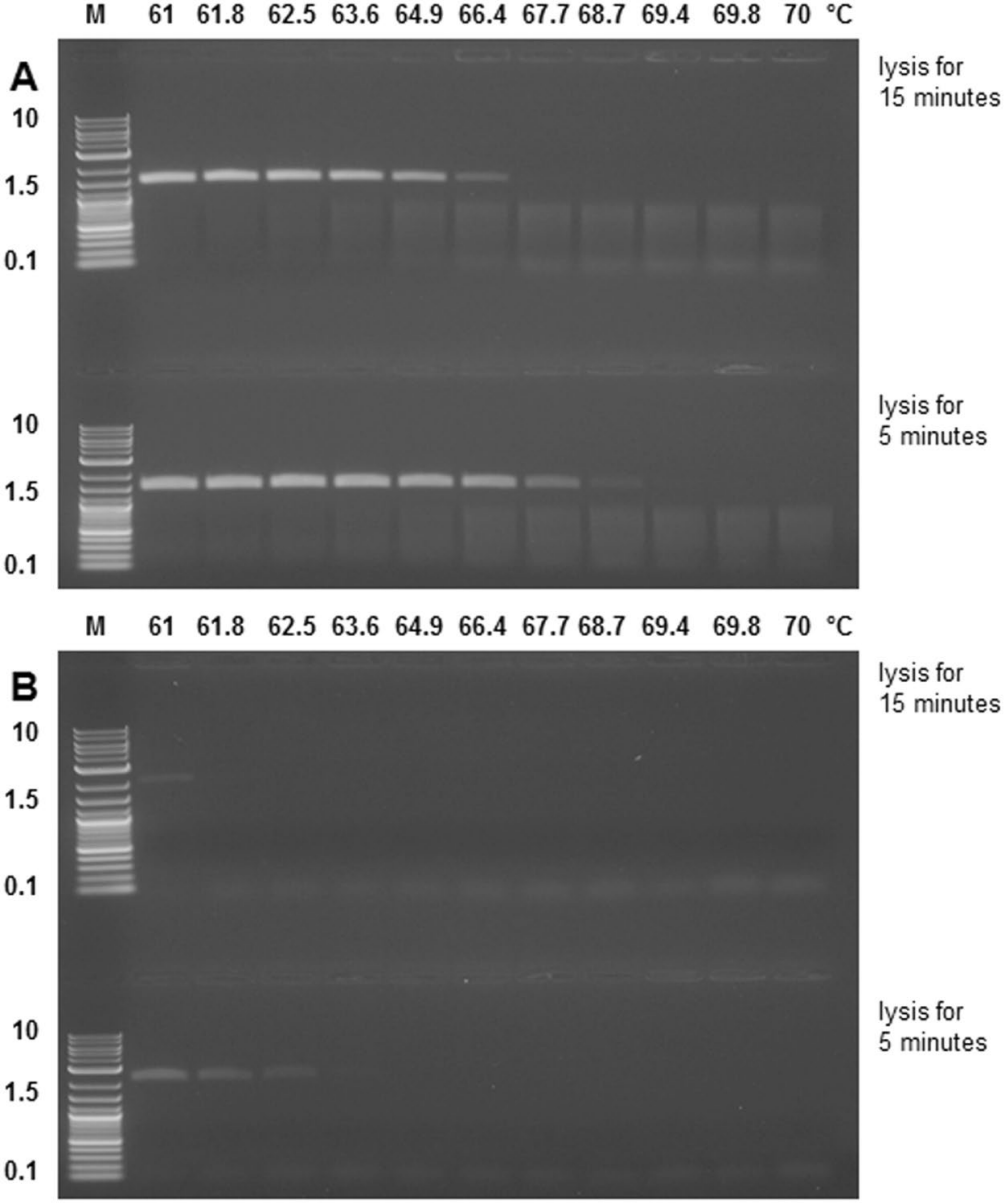
Temperature stability testing of particles. (**A**) Temperature stability testing of MS2 phage-like particles (MS2 PLP) and (**B**) His-tagged MS2 phage-like particles (His-tagged MS2 PLP) using agarose gel electrophoresis; temperature values are in °C; M, marker, 2-log DNA ladder (New England Biolabs, UK); the marker values are in kb. The figures were cropped, full length gels are presented presented in Supplementary Figures 6 and 7.

The lysis temperature of MS2 PLP and His-tagged MS2 PLP was determined as the temperature at which no band was readily visible on the agarose gel. The MS2 PLP had higher temperature stability (lysis temperature 66.4 °C/15 minutes, 68.7 °C/5 minutes; Fig. 6A) than the His-tagged MS2 PLP (61 °C/15 minutes, 62.5 °C/5 minutes; Fig. 6B).

### Determination of optimal method of thermal lysis of His-tagged MS2 PLP and comparison of quantity and purity of MS2 PLP and His-tagged MS2 PLP

The RT-qPCR results showing the most suitable conditions for thermal lysis of His-tagged MS2 PLP are summarized in Table 1 (Table 1). Based on these results, it was decided to include the step of particle lysis into the step of initial denaturation in RT protocol. The RT-qPCR results comparing the quantity and purity of MS2 PLP and His-tagged MS2 PLP are summarized in Table 2 (Table 2).

**Table 1:** RT-qPCR testing of optimal method of thermal lysis of His-tagged MS2 phage-like particles (His-tagged MS2 PLP).

Lysis temperature (°C)	Mean concentration of His-tagged MS2 PLP (GE/µl) ± SD^1^	Positive samples/analyzed samples
65/5 min	5.90 × 10^6^ ± 5.26 × 10^5^	8/8
75/5 min	7.03 × 10^6^ ± 5.53 × 10^5^	8/8
85/5 min	8.62 × 10^6^ ± 1.33 × 10^6^	8/8
95/5 min	5.83 × 10^6^ ± 5.80 × 10^5^	8/8
65/5 min in RT step	8.24 × 10^7^ ± 6.69 × 10^6^	8/8

^1^Total amount of His-tagged MS2 PLP from each sample was determined according to the calibration curve included in each RT-qPCR experiment. SD, standard deviation. GE, genomic equivalents.

**Table 2:** The results of RT-qPCR purity testing of His-tagged MS2 phage-like particles (His-tagged MS2 PLP) and MS2 phage-like particles (MS2 PLP).

MS2 PLP^1^		Contaminating DNA^2^		
Concentration (GE/µl) ± SD	Positive/analyzed	Concentration (GE/µl) ± SD	Positive/analyzed	%
**His-tagged MS2 PLP**				
0.63 × 10^8^ ± 8.93 × 10^6^	8/8	6.55 × 10^1^ ± 1.47 × 10^1^	8/8	0.00010
1.93 × 10^7^ ± 2.64 × 10^6^	8/8	6.26 × 10^0^ ± 5.99 × 10^0^	8/8	0.00003
1.00 × 10^6^ ± 6.91 × 10^4^	8/8	2.43 × 10^0^ ± 3.25 × 10^0^	3/8	0.00024
1.01 × 10^5^ ± 1.05 × 10^4^	8/8	0	0/8	0.00000
**Wild type MS2 PLP**				
0.66 × 10^8^ ± 9.21 × 10^6^	8/8	6.46 × 10^7^ ± 9.19 × 10^6^	8/8	97.87000
0.97 × 10^7^ ± 1.11 × 10^6^	8/8	6.85 × 10^6^ ± 3.93 × 10^6^	8/8	70.61000
1.11 × 10^6^ ± 2.67 × 10^5^	8/8	4.07 × 10^5^ ± 1.82 × 10^5^	8/8	36.66000
1.92 × 10^5^ ± 9.18 × 10^4^	8/8	3.73 × 10^4^ ± 1.55 × 10^4^	8/8	19.42000

GE – genomic equivalents.

^1^Total amount of His-tagged MS2 PLP and MS2 PLP from each sample was determined according to the calibration curve included in each RT-qPCR experiment.

^2^Total amount of contaminating DNA was determined according to the calibration curve included in each RT-qPCR experiment (RT enzyme not included).

## Discussion

It was shown previously that the newest and most advanced *in vivo* system for production of MS2 PLP–the one-plasmid double-expression system–has great potential for the production of MS2 PLP^11,12^. In comparison with other previously used systems, the system developed by Zhan *et al*. has a good expression efficiency, produces MS2 PLP containing only defined control ssRNA sequences without any other MS2 bacteriophage sequences, and also allows packaging of long control ssRNA sequences using multiple *pac* sites^10–12^. However, the one-plasmid double-expression system does not enable rapid, simple, and effective purification of produced MS2 PLP. This problem can be resolved by introduction of a His-tag into the structure of MS2 PLP. This results in MS2 PLP carrying 180 His-tag epitopes, which allows their purification using affinity chromatography^13^. Unfortunately, at the beginning of this study it was found that introduction of a His-tag into the structure of MS2 PLP according to Cheng *et al*. and expression of His-tagged MS2 PLP in a one-plasmid double-expression system does not result in the production of any His-tagged MS2 PLP^13^. It was subsequently discovered that another group also encountered this problem and failed in the production of His-tagged MS2 PLP (Harder, Friedrich Loeffler Institute, Germany, personal communication, March 2017). Ponchon *et al*. also adopted the previously reported strategy of Cheng *et al*. and tried to produce His-tagged MS2 PLP^15,16^. However, Ponchon *et al*. used a so-called two-plasmid system where the first plasmid carried the MS2 coat protein sequence under control of an inducible tac promoter and the second plasmid carried the sequence for packaging under the control of a constitutive lpp promoter^10,16^. This strategy allows successful co-expression of both the protein and ssRNA in an appropriate ratio. But as a result, in all cases, it was observed that the MS2 coat protein with the His-tag only formed a dimer rather than intact His-tagged MS2 PLP^16^. It is known that peptide insertions into viral capsid proteins often result in protein misfolding that interferes with MS2 PLP assembly^17^. On the other hand, it is also known that fusion of two coat protein monomers (single-chain dimer) confers tolerance to peptide insertions in an MS2 coat protein dimer^18,19^. The basic idea of a using a single-chain version of the MS2 coat protein dimer with a His-tag in this study was inspired by these previously published results. The described plasmid-driven system is based on plasmid pACYCDuet-1. Plasmid pACYCDuet-1 was chosen as the basis for the new His-tagged MS2 PLP production system because it was shown to be an optimal solution in previous studies that dealt with the construction of particles or production of MS2 coat protein^11,20–22^. Zhan *et al*. used a one-plasmid double-expression system based on the pACYCDuet-1 plasmid for production of MS2 PLP carrying a 3,034-nt-long control sequence^11^. To test the ability of such a system to also pack very short control sequences, our previously reported specific control sequence with a total length of 150 nt was chosen for packaging^8^. The final pACYCDuet-1-TM-CoatDimer-His expression vector was constructed with a view of easily allowing subsequent modifications. MCS1 of the vector containing the sequences of maturase and single-chain version of the coat protein dimer containing the His-tag was modified, so that the maturase sequence no longer contains the *Nde*I restriction site. This modification opens the possibility for using the *Nde*I restriction site for cloning into MCS2, which increases the maximum length of packed ssRNA control sequences, because *Nde*I is the first restriction site in the MCS2 of the vector. Furthermore, in the case that the *Nde*I sequence will be present in the sequence of maturase, direct cloning of sequences into MCS2 of the vector using the *Nde*I restriction site will not be possible. The inserted sequence for the His-tag is flanked by two *Kpn*I restriction sites. This flanking is a consequence of previously used methods of oligonucleotide-directed mutagenesis^14,23^. Although the sequence of maturase and the single-chain version of coat protein dimer containing the His-tag was synthesized *de novo*, it was decided to maintain the presence of the *Kpn*I restriction sites, mainly due to the possibility of easy replacement of the His-tag sequence at this position. The single-chain version of the coat protein dimer is more tolerant of foreign peptide insertions than the wild-type coat protein monomer^18,19^. Therefore, utilization of the described system should allow insertion of different peptides at this position without causing any problems for MS2 PLP assembly. The possibility of producing MS2 PLP carrying ssRNAs of choice and presenting various peptides on the surface of the capsid is currently a very promising method for the production of RNA vaccines based on recombinant MS2 PLP^24–28^.

The single-chain version of the MS2 coat protein dimer contains one wild-type coat protein fused with one mutagenized coat protein carrying the His-tag. Therefore, the presented method results in the production of His-tagged MS2 PLP containing 90 His-tags in the capsid structure exposed outward from the particle and allowing chelated Co^2+^ on the resin beads to access the His-tag. Newly produced single-chain version coat protein dimer containing the His-tag was extensively characterized using SDS-PAGE, western blotting and MALDI-TOF analyses. The molecular weight of the wild-type coat protein measured using MALDI-TOF was 13,730 Da, which is very close to its theoretical value of 13,728.54 Da. The measured molecular weight of the single-chain version of the coat protein dimer containing the His-tag was 28,329 Da (Fig. 4E), which is also very close to its theoretical value of 28,333 Da. The differences between theoretical and measured values of protein molecular weights are probably due to the sample preparation protocol and instrumentation used. These analyses clearly showed that the desired mutagenized coat protein with the expected properties was successfully obtained after induction. Moreover, this single-chain version of the coat protein dimer containing the His-tag was able to create compact His-tagged MS2 PLP, which were morphologically indistinguishable from previously produced MS2 PLP^2,8^.

The presence of the His-tag in the structure of the coat protein was used for purification of His-tagged MS2 PLP through FPLC. In comparison with the traditionally used method of ultracentrifugation^4,8,11^, the FPLC approach is a rapid purification technique enabling the isolation of huge quantities of His-tagged MS2 PLP (~10^10^ particles/µl; RT-qPCR quantification according to^8^) in less than one hour. In fact, the quantity of isolated His-tagged MS2 PLP purified through FPLC is one order of magnitude higher than the quantity of MS2 PLP purified through ultracentrifugation (~10^9^ particles/µl; RT-qPCR quantification according to^8^). The FPLC purification procedure is also not as laborious as traditional methods of purification and does not have any negative impact on MS2 PLP integrity. The integrity of His-tagged MS2 PLP after FPLC purification was tested by TEM; the stability of particles against nucleases was also tested. In general, the ability of MS2 PLP to withstand the action of nucleases is one of their most important properties, because only intact particles are able to protect the ssRNA that they encapsulate. Testing the stability of FPLC-purified His-tagged MS2 PLP against nucleases clearly showed that the particles were able to effectively protect the ssRNA that they encapsidated against nuclease degradation.

It was shown previously that MS2 PLP consisting of a single-chain version of the coat protein dimer have lower temperature stability in comparison with MS2 PLP consisting of a wild-type coat protein dimer^29,30^. Our results indicate that lysis temperature is a function of quantity, time, and temperature. It is also interesting that the intensity of bands on an agarose gel was much higher in the case of MS2 PLP in comparison with His-tagged MS2 PLP, although the quantity of tested particles was the same–0.7 µg per band, which corresponds approximately to 10^12^ particles^8^. A possible explanation for this difference is that the lower intensity of His-tagged MS2 PLP bands is related to a lower proportion of contaminating DNA in comparison with MS2 PLP (Table 2).

Empirically, the lysis temperature of His-tagged MS2 PLP was determined to be 62.5 °C/5 min (Fig. 6). This feature was utilized in our previously developed RT-qPCR system for quantification of MS2 PLP, which uses a two-step format in which RT and qPCR reactions are separated^8^. The step of particle lysis was included in the RT protocol and it was demonstrated that initial denaturation of 65 °C/5 minutes during the RT protocol is sufficient to lyse His-tagged MS2 PLP. This approach not only saves time, but also represents the gentlest method of thermal lysis and provides the highest RT-qPCR yield. On the other hand, the results of this experiment also show that the temperature of lysis has no critical effect on ssRNA stability, and, thus, the STE buffer in which the particles were diluted and lysed provided good protection to naked ssRNA molecules.

The purity of produced MS2 PLP is essential, and, therefore, elimination of contaminating *E*. *coli* genomic DNA from lysed bacterial culture prior to MS2 PLP purification is a part of each production protocol; usually, a mixture of DNases and RNases are used^2,3,5,8,11,12^. It is generally accepted that this treatment is sufficient to eliminate contaminating DNA. However, this may not be true. Cheng *et al*. tested the purity of MS2 PLP purified through ultracentrifugation and found that they exhibit significant DNA contamination^13^. On the other hand, His-tagged MS2 PLP exhibit only negligible DNA contamination (but the determined quantity of His-tagged MS2 PLP was only ~10^3^ particles/µl). Based on these findings, the His-tagged MS2 PLP produced in this study and previously produced MS2 PLP^8^ were tested for the presence of contaminating DNA. Because some MS2 PLP applications, e.g., use as a PCV in RT-qPCR detection and quantification of pathogenic ssRNA viruses from a wide range of samples, require a greater quantity of MS2 PLP (~10^5^–10^8^ particles/µl), the presence of contaminating DNA was tested in range of 10^8^–10^5^ particles/µl. The results are consistent with those reported previously^13^ with the difference that His-tagged MS2 PLP are also not absolutely free of contaminating DNA, especially at higher concentrations. On the other hand, according to our findings His-tagged MS2 PLP are always at least 150,000× cleaner through all tested concentrations (10^8^–10^5^ particles/µl) than MS2 PLP purified through ultracentrifugation (Table 2). Moreover, the proportion of contaminating DNA decreases with progressive dilution, but only His-tagged MS2 PLP can be diluted to a quantity where contaminating DNA is not detectable in RT-qPCR. In general, the purity of isolated MS2 PLP is very important because the presence of contaminating DNA could affect quantification in experiments where particles are used as a PCV, or can confer toxic effects on MS2 PLP serving as vaccines or drug delivery systems^9^. Thus, the one-plasmid double-expression His-tag system presented in this work is able to meet this demanding criterion, and can be used for the production of highly pure MS2 PLP. On the other hand, the described purification method is dedicated mainly for using MS2 PLP as a PCV and for using as a vaccines or drug delivery systems it is needed to perform additional laboratory tests.

## Methods

### Construction of specific control sequence

A specific control sequence derived from mtDNA sequences of two extinct species–thylacine (*Thylacinus cynocephalus*) and the moa bird (*Dinornis struthoides*)–was constructed as described previously^8^.

### Construction of pACYCDuet-1-TM

The specific control sequence was cloned into multiple cloning site 2 (MCS2) of the pACYCDuet-1 vector (p15A-type replication origin; Novagen, Merck, Germany; Fig. 1). In brief, a 191-bp amplicon encoding the specific control sequence was obtained by PCR using a *de novo* template construct and the TM-NdeI F and TM-AvrII pac R primer pair, which included *Nde*I and *Avr*II restriction enzyme sites; the TM-AvrII pac R primer also included one C-variant *pac* site (Table 3). The reaction mixture was composed of 12.5 µl of FastStart PCR master (Roche Molecular Diagnostics, Germany), 7.5 pmol of each primer and 3.3 pmol of template DNA. The assay was run in a total volume of 25 µl under the following conditions: 95 °C for 3 minutes, followed by 25 cycles of 94 °C for 30 seconds, 56 °C for 30 seconds, and 72 °C for 30 seconds; final extension was at 72 °C for 5 minutes. The results of the PCR amplification were examined by agarose gel electrophoresis (1%). The PCR product was purified using the QIAquick PCR purification kit (Qiagen, Germany) and subsequently cleaved at 37 °C for 2 hours. The composition of the restriction mixture was 500 ng of PCR product, 2 µl of NEBuffer 2 (New England Biolabs, UK; NEB), 20 U and 4 U of *Nde*I and *Avr*II endonucleases, respectively (both NEB), in a final volume of 20 µl. The cleaved PCR product was also purified using the QIAquick PCR purification kit (Qiagen).

**Table 3:** Specific oligonucleotides used in present study.

Name	Sequence
TM NdeI F	5′-GATACACATATGCGCTTCCGTCAAACCCCTAA-3′
TM AvrII pac R	5′-TAGGCCTAGG**ACATGGGTGATCCTCATGT**GGGTTTAGAATGTTTTCTCCCGT-3′
TM F	5′-CGCTTCCGTCAAACCCCTAA-3′
TM R	5′-GGTTTAGAATGTTTTCTCCCGT-3′
DuetUP2^1^	5′-TTGTACACGGCCGCATAATC-3′
T7 terminator^1^	5′-GCTAGTTATTGCTCAGCGG-3′
DuetUP1^1^	5′-GGATCTCGACGCTCTCCCT-3′
DuetDOWN1^1^	5′-GATTATGCGGCCGTGTACAA-3′
MS2 NcoI	5′-ATGCCCATGGTGGCTATCGCTGTAGGTAGCC-3′
MS2 NotI	5′-AAGGAAAAAAGCGGCCGCTGGCCGGCGTCTATTAGTAG-3′

^1^Sequences of oligonucleotides was designed according to pACYCDuet-1 manufacturer’s instructions.

Underlined sequences are *Nde*I or *Avr*II, *Nco*I and *Not*I restriction enzyme sites. The sequence in bold is the C-variant *pac* site.

The pACYCDuet-1 vector was cleaved at 37 °C for 2 hours. The restriction mixture was composed of 500 ng of plasmid DNA, 5 µl of NEBuffer 2 (NEB) 20 U and 4 U of *Nde*I and *AvrII* endonucleases, respectively (both NEB), in a final volume of 50 µl. The cleaved vector was dephosphorylated with 0.25 U of calf-intestinal alkaline phosphatase (CIP) (NEB) at 37 °C for 1 hour. Subsequently, vector DNA was purified using the QIAquick PCR purification kit (Qiagen).

Ligation of the specific control sequence to the MCS2 of the cleaved pACYCDuet-1 vector was performed using the Quick-ligation kit (NEB) according to the manufacturer’s instructions. Transformation of *E*. *coli* TOP10 (Life Technologies, USA) was also performed according to the manufacturer’s recommendations. Transformed cells were grown on Luria-Bertani (LB) (Sigma-Aldrich, Czech Republic) agar plates containing chloramphenicol (34 µg/ml, Sigma-Aldrich) overnight. Plasmid DNA was isolated with the NucleoSpin Plasmid kit (Macherey-Nagel, Germany) according to the manufacturer’s instructions. The presence of specific inserts was confirmed by vector-specific (DuetUP2 and T7 terminator) and insert-specific (TM F and TM R) primers (Table 3) in PCRs as well as by sequencing (Eurofins MWG Operon, Germany).

### Construction of the expression vector with a single-chain version of the coat protein dimer containing the His-tag for production of His-tagged MS2 PLP - pACYCDuet-1-TM-CoatDimer-His

The sequence of maturase and the single-chain version of the coat protein dimer containing the His-tag were synthesized *de novo* (Life Technologies) (Fig. 2). The *de novo* construct also contained a transversion of adenine (**A**) at position 639 to cytosine (**C**). This change does not influence the amino acid sequence of maturase but leads to deletion of the *Nde*I restriction site (C**A**TATG −› C**C**TATG). Fusion of the two sequences encoding the coat protein was done as in the pCT2dl-13 construct^18,19^. The sequence (-GGTACCCATCACCATCACCATCACGGTACC-) encoding the His-tag flanked by two *Kpn*I restriction sites (underlined)^13^ was inserted between residues 11 and 12 of the second coat protein amino acid sequence (corresponding to the position between codons 15 and 16 of wild-type coat protein sequence)^14^. The *de novo* construct was verified by sequencing (Life Technologies).

The fragment encoding the maturase and single-chain version of the coat protein dimer with the His-tag modification was obtained by PCR with the KAPA HiFi HotStart PCR Kit (Kapa Biosystems, USA) using the *de novo* template construct and the MS2 NcoI and MS2 NotI primer pair, which included *Nco*I and *Not*I restriction enzyme sites (Table 1). The reaction mixture was composed of 5 µl of 5 × Kapa HiFi fidelity buffer, 0.3 mM dNTP mix, 7.5 pmol of each primer, 1 U of Kapa HiFi polymerase, and 5 pmol of template DNA. The assay was run in a total volume of 25 µl under the following conditions: 95 °C for 5 minutes, followed by 25 cycles of 98 °C for 20 seconds, 55 °C for 15 seconds, and 72 °C for 70 seconds; final extension was at 72 °C for 5 minutes. The results of the PCR amplification were examined by agarose gel electrophoresis (1%). The PCR product was purified using the QIAquick PCR purification kit (Qiagen) and subsequently cleaved at 37 °C for 2 hours. The restriction mixture was composed of 500 ng of PCR product, 2 µl of NEBuffer 3 (NEB), and 10 U each of *Nco*I and *Not*I endonucleases (both NEB), in a final volume of 20 µl. The cleaved PCR product was purified using the QIAquick PCR purification kit (Qiagen).

The pACYCDuet-1-TM vector was cleaved at 37 °C for 2 hours. The restriction mixture was composed of 500 ng of plasmid DNA, 5 µl of NEBuffer 3 (NEB), and 10 U each of *Nco*I and *Not*I endonucleases (both NEB), in a final volume of 50 µl. Cleaved vector was dephosphorylated using 0.25 U CIP (NEB) at 37 °C for 1 hour. Subsequently, vector DNA was purified using the QIAquick PCR purification kit (Qiagen).

Ligation of the fragment encoding the maturase and single-chain version of the coat protein dimer with the His-tag modification to the MCS1 of the pACYCDuet-1-TM vector was performed using the Quick-ligation kit (NEB) according to the manufacturer’s instructions. Transformation of *E*. *coli* TOP10 (Life Technologies) was also performed according to the manufacturer’s recommendations. Transformed cells were grown on LB (Sigma-Aldrich) agar plates containing chloramphenicol (34 µg/ml) overnight. Plasmid DNA was isolated with the NucleoSpin Plasmid kit (Macherey-Nagel) according to the manufacturer’s instructions. The presence of specific inserts was confirmed by vector-specific (DuetUP1 and DuetDOWN1) and insert-specific (MS2 NcoI and MS2 NotI) primers (Table 3) in PCRs as well as by sequencing (Eurofins MWG Operon).

### Induction of expression of His-tagged MS2 PLP

The constructed pACYCDuet-1-TM-CoatDimer-His plasmid was transformed into *E*. *coli* BL21 (DE3) cells (NEB) according to the manufacturer’s instructions and the bacterial cells were grown in LB broth (Sigma-Aldrich) containing chloramphenicol (34 µg/ml) at 37 °C until OD_600_ = 1.7. Two ml of the bacterial culture were transferred to 200 ml of Terrific broth (TB) medium (24 g/l yeast extract, 20 g/l tryptone, 4 ml/l glycerol, 0.017 M KH_2_PO_4_, 0.072 M K_2_HPO_4_, all Sigma-Aldrich) containing chloramphenicol (34 µg/ml) and cultivated at 37 °C until OD_600_ = 0.8 and centrifuged at 6000 × g for 10 minutes at 25 °C. Subsequently, the pellet was resuspended in 200 ml of fresh TB medium containing chloramphenicol (34 µg/ml). Protein expression was induced by addition of 2 mM isopropyl-L-thio-D-galactopyranoside (IPTG) (Sigma-Aldrich) to the culture, after 30 minutes of culture growth at 37 °C, rifampicin (Sigma-Aldrich) was added to the IPTG induced culture to a final concentration of 200 µg/ml and the culture was cultivated at 37 °C for 16 hours.

The cell suspension was centrifuged at 6000 × g for 10 minutes at 4 °C and cells were washed and centrifuged (6000 × g for 10 minutes at 4 °C) twice in 200 ml of PBS buffer (pH = 7.2). The pellet was resuspended in 4 ml of sonication buffer (50 mM Tris, 5 mM MgCl_2_ × 6H_2_O, 5 mM CaCl_2_, 0.1 M NaCl, pH = 8.0, all Sigma-Aldrich) and 200 U of Turbo DNase (Ambion, ThermoFisher Scientific, USA), 700 U of RNase A (Qiagen), and 2500 U of benzonase nuclease (Sigma-Aldrich) were added to eliminate *E*. *coli* genomic DNA and RNA. The cells were lysed by ultrasonic disruption (Bandelin VW3100 sonicator, probe MS73, Bandelin, Germany) at amplitude 50%, using four pulses for a total length of 2 minutes at 4 °C. Sonicated bacterial suspension was incubated at 37 °C for 3 hours. To eliminate cell debris, the lysed bacterial suspension was briefly centrifuged at 6000× g for 15 minutes at room temperature and the supernatant containing His-tagged MS2 PLP was filtered through a 0.22-µm syringe PES filter (TPP, Switzerland).

### Purification of His-tagged MS2 PLP

Filtered supernatant containing His-tagged MS2 PLP was mixed in 1:1 ratio with 2 × concentrated 0.22-µm syringe PES (TPP)-filtered binding buffer (100 mM NaH_2_PO_4_, 600 mM NaCl, 30 mM imidazole, pH = 8.0, all Sigma-Aldrich). Subsequently, His-tagged MS2 PLP were purified using a fast performance liquid chromatography system (FPLC; Pharmacia, Sweden) with a HiTrap TALON Crude (5 ml) column (GE Healthcare, UK). In brief, the column was preequilibrated with 50 ml of 0.22-µm (TPP)-filtered binding buffer (50 mM NaH_2_PO_4_, 300 mM NaCl, 15 mM imidazole, pH = 8.0, all Sigma-Aldrich) at a flow rate of 10 ml/min. The His-tagged MS2 PLP bound to the column (flow rate 1 ml/min) and were subsequently washed with 200 ml of 0.22-µm (TPP)-filtered washing buffer (50 mM NaH_2_PO_4_, 300 mM NaCl, 30 mM imidazole, pH = 8.0, all Sigma-Aldrich) at a flow rate of 10 ml/min. His-tagged MS2 PLP were eluted from the column in a final volume of 5 ml with 0.22-µm (TPP)-filtered elution buffer (50 mM NaH_2_PO_4_, 300 mM NaCl, 200 mM imidazole, pH = 8.0, all Sigma-Aldrich) at a flow rate of 0.5 ml/min. Eluate containing His-tagged MS2 PLP was desalted using Vivacon 10 kDa columns (Vivacon, Sartorius, Germany) and converted to STE buffer (10 mM Tris, 100 NaCl, 1 mM EDTA, pH = 7.5, all Sigma-Aldrich). His-tagged MS2 PLP were verified by TEM using a Philips EM 208 electron microscope (FEI, Czech Republic). The sample of supernatant containing His-tagged MS2 PLP was applied to the grid, stained with 2% ammonium molybdate (pH = 7.0) for 1 minute, and inspected at 18,000 × magnification and an accelerating voltage of 80 kV. Purified His-tagged MS2 PLP were quantified by RT-qPCR as was described previously^8^. The quantity of FPLC-purified His-tagged MS2 PLP was compared with previously produced non-His-tagged MS2 PLP purified through ultracentrifugation^8^.

### Characterization of the single-chain version of the coat protein dimer containing the His-tag

To confirm the production of the single-chain version of the coat protein dimer containing the His-tag, SDS-PAGE, western blot analysis, and MALDI-TOF analysis were applied. As a control material, previously produced MS2 PLP without His-tag, composed of wild-type MS2 bacteriophage coat protein^8^, were used. Briefly, MS2 PLP and His-tagged MS2 PLP were resolved by SDS-PAGE^31^ using a 15% separating polyacrylamide gel and transferred to polyvinylidene difluoride membranes (PVDF; Amersham, UK) which were subsequently blocked in 1% casein hydrolysate (Imuna, Czech Republic) overnight at 4 °C. Protein bands resolved by SDS-PAGE were visualized by staining with Coomassie brilliant blue R-250. The membranes were incubated with Anti-Enterobacterio Phage MS2 Coat Protein polyclonal antibody (Merck Millipore, USA) at a dilution of 1:1500 for 1 hour at room temperature, washed in PBS with 0.05% Tween-20 (PBS/T, Serva, Germany), and then incubated for 1 hour at room temperature with goat anti-rabbit IgG conjugated with HRP diluted 1:1500 (Jackson ImmunoResearch, UK). After a washing step, the protein bands were visualized with 3,3′-diaminobenzidine (Sigma-Aldrich). Western blotting with an anti-HisTag antibody (Pierce, Thermo Scientific, USA) diluted 1:3000 as primary antibody and goat anti-mouse IgG conjugated with HRP diluted 1:1000 (Jackson ImmunoResearch) as a secondary antibody was performed as described above.

MALDI-TOF analysis was performed as follows: MS2 PLP^8^ and His-tagged MS2 PLP were treated with 10% β-mercaptoethanol (Sigma-Aldrich) at 95 °C for 1 minute and precipitated at 0 °C for 15 minutes. Precipitated proteins were resuspended in 1% trifluoroacetic acid and desalted using 3 kDa and 10 kDa Vivacon columns (Sartorius). MALDI-TOF MS analysis was carried out using an Ultraflextreme instrument (Bruker Daltonics, USA) operated in linear positive detection mode. Ferulic acid (Sigma-Aldrich) (12.5 mg/ml in water:acetonitrile:formic acid, 50:33:17, v/v mixture) was used as the MALDI matrix in combination with a stainless steel sample plate.

### Stability of His-tagged MS2 PLP against nucleases

Stability of His-tagged MS2 PLP against nucleases was verified by their incubation with the combination of 10 U of Turbo DNase (Ambion) and/or 10 U of RNase A (Qiagen) at 37 °C for 1 hour. As controls for the reaction, DNA (500 ng of purified PCR fragment encoding the maturase and single-chain version of the coat protein dimer with His-tag modification) and RNA (500 ng of IAC *in vitro* transcript^8^), were added to the tested His-tagged MS2 PLP. The ability of His-tagged MS2 PLP to resist the impact of nucleases was determined using agarose gel electrophoresis (0.8%).

### Temperature stability of MS2 PLP and His-tagged MS2 PLP

MS2 PLP^8^ and His-tagged MS2 PLP (both 7 µg, ~10^12^ particles) dissolved in STE buffer were incubated for 15 minutes and 5 minutes, respectively, at the selected temperatures. The BioRad Tetrad 2 Peltier Thermal Cycler (BioRad, USA) temperature gradient program (61 °C –70 °C) was used. Immediately after heating, the samples were loaded onto 1% agarose gel and the particle position was visualized under UV illumination.

### Determination of optimal method of thermal lysis of His-tagged MS2 PLP and comparison of quantity and purity of MS2 PLP and His-tagged MS2 PLP

Based on the results of temperature stability testing, His-tagged MS2 PLP particles were diluted to an approximate concentration of 10^6^ particles/µl. The influence of lysis temperature on the exact quantity of His-tagged MS2 PLP was tested using a previously described quantitative reverse transcription polymerase chain reaction (RT-qPCR) system^8^ with small modifications; non-treated and thermally treated (65 °C, 75 °C, 85 °C and 95 °C for 5 minutes) His-tagged MS2 PLP were added directly to the reverse transcription (RT) reaction. RT was carried out in octuplicates for each temperature and qPCR was done in duplicate for each sample.

Subsequently, the quantity and purity of His-tagged MS2 PLP was compared with the quantity and purity of previously produced MS2 PLP^8^. MS2 PLP were thermally lysed according to the original protocol (95 °C for 5 minutes), while His-tagged MS2 PLP were added directly at the RT step without undergoing thermal lysis according to the results of determination of the optimal method of thermal lysis. The quantity of tested particles was in the range of 10^8^–10^5^ particles/µl. To determinate the precise amount of contaminating DNA in the sample, RT-qPCR amplification of the specific control sequence was done using reactions with and without RT enzyme (the enzyme was replaced with RNase-free H_2_O; Top-Bio, Czech Republic). RT was carried out in octuplicates for each set of samples and qPCR was done in duplicate for each sample.

## Electronic supplementary material

Supplementary information

## Data Availability

The datasets generated during and/or analysed during the current study are available from the corresponding author on reasonable request.
