# Author Correction: One-plasmid double-expression His-tag system for rapid production and easy purification of MS2 phage-like particles

**DOI:** 10.1038/s41598-019-39693-2

**Published:** 2019-04-17

**Authors:** Pavel Mikel, Petra Vasickova, Petr Kralik

**Affiliations:** 1 Veterinary Research Institute, Hudcova 296/70, 621 00 Brno, Czech Republic; 2 Department of Experimental Biology, Faculty of Science, Masaryk University, Kamenice 753/5, 625 00 Brno, Czech Republic

Correction to: *Scientific Reports* 10.1038/s41598-017-17951-5, published online 13 December 2017

In the original version of the Supplementary File which accompanied this Article, the plasmid sequence was provided as a flat image. The file has now been replaced: the corrected version contains the full sequence of the plasmid used in this study in a machine readable format.

