# Supplementary material for: One-plasmid double-expression His-tag system for rapid production and easy purification of MS2 phage-like particles
Source: Sci Rep. 2017 Dec 13;7:17501. doi: 10.1038/s41598-017-17951-5 (PMC5727534; doi:10.1038/s41598-017-17951-5)
Supplement: Supplementary file 1 — Supplementary information [file 41598_2017_17951_MOESM1_ESM.docx]

**One-plasmid double-expression His-tag system for rapid production and easy purification of MS2 phage-like particles**

Pavel Mikel*, Petra Vasickova and Petr Kralik

A complete sequence of the expression vector with a single-chain version of the coat protein dimer containing the His-tag for production of His-tagged MS2 PLP - pACYCDuet-1-TM-CoatDimer-His

ggggaattgtgagcggataacaattcccctgtagaaataattttgtttaactttaataaggagatata**ccatgg**TGGCTATCGCTGTAGGTAGCCGGAAT 100

TCCATTCCTAGGAGGTTTGACCTGTGCGAGCTTTTAGTACCCTTGATAGGGAGAACGAGACCTTCGTCCCCTCCGTTCGCGTTTACGCGGACGGTGAGAC 200

TGAAGATAACTCATTCTCTTTAAAATATCGTTCGAACTGGACTCCCGGTCGTTTTAACTCGACTGGGGCCAAAACGAAACAGTGGCACTACCCCTCTCCG 300

TATTCACGGGGGGCGTTAAGTGTCACATCGATAGATCAAGGTGCCTACAAGCGAAGTGGGTCATCGTGGGGTCGCCCGTACGAGGAGAAAGCCGGTTTCG 400

GCTTCTCCCTCGACGCACGCTCCTGCTACAGCCTCTTCCCTGTAAGCCAGAACTTGACTTACATCGAAGTGCCGCAGAACGTTGCGAACCGGGCGTCGAC 500

CGAAGTCCTGCAAAAGGTCACCCAGGGTAATTTTAACCTTGGTGTTGCTTTAGCAGAGGCCAGGTCGACAGCCTCACAACTCGCGACGCAAACCATTGCG 600

CTCGTGAAGGCGTACACTGCCGCTCGTCGCGGTAATTGGCGCCAGGCGCTCCGCTACCTTGCCCTAAACGAAGATCGAAAGTTTCGATCAAAACACGTGG 700

CCGGCAGGTGGTTGGAGTTGCAGTTCGGTTGGTTACCACTAATGAGTGATATCCAGGGTGC**C**TATGAGATGCTTACGAAGGTTCACCTTCAAGAGTTTCT 800

TCCTATGAGAGCCGTACGTCAGGTCGGTACTAACATCAAGTTAAATGGCCGTCTGTCGTATCCAGCTGCAAACTTCCAGACAACGTGCAACATATCGCGA 900

CGTATCGTGATATGGTTTTACATAAACGATGCACGTTTGGCATGGTTGTCGTCTCTAGGTATCTTGAACCCACTAGGTATAGTGTGGGAAAAGGTGCCTT 1000

TCTCATTCGTTGTCGACTGGCTCCTACCTGTAGGTAACATGCTCGAGGGCCTTACGGCCCCCGTGGGATGCTCCTACATGTCAGGAACAGTTACTGACGT 1100

AATAACGGGTGAGTCCATCATAAGCGTTGACGCTCCCTACGGGTGGACTGTGGAGAGACAGGGCACTGCTAAGGCCCAAATCTCAGCCATGCATCGAGGG 1200

GTACAATCCGTATGGCCAACAACTGGCGCGTACGTAAAGTCTCCTTTCTCGATGGTCCATACCTTAGATGCGTTAGCATTAATCAGGCAACGGCTCTCTA 1300

GATAGAGCCCTCAACCGGAGTTTGAAGCATGGCTTCTAACTTTACTCAGTTCGTTCTCGTCGACAATGGCGGAACTGGCGACGTGACTGTCGCCCCAAGC 1400

AACTTCGCTAACGGGGTCGCTGAATGGATCAGCTCTAACTCGCGTTCACAGGCTTACAAAGTAACCTGTAGCGTTCGTCAGAGCTCTGCGCAGAATCGCA 1500

AATACACCATCAAAGTCGAGGTGCCTAAAGTGGCAACCCAGACTGTTGGTGGTGTAGAGCTTCCTGTAGCCGCATGGCGTTCGTACTTAAATATGGAACT 1600

AACCATTCCAATTTTCGCTACGAATTCCGACTGCGAGCTTATTGTTAAGGCAATGCAAGGTCTCCTAAAAGATGGAAACCCGATTCCCTCAGCAATCGCA 1700

GCAAACTCCGGCATCTACGCTAACTTTACTCAGTTCGTTCTCGTCGACAATGGCGGTACCCATCACCATCACCATCACGGTACCGGCGACGTGACTGTCG 1800

CCCCAAGCAACTTCGCTAACGGGGTCGCTGAATGGATCAGCTCTAACTCGCGTTCACAGGCTTACAAAGTAACCTGTAGCGTTCGTCAGAGCTCTGCGCA 1900

GAATCGCAAATACACCATCAAAGTCGAGGTGCCTAAAGTGGCAACCCAGACTGTTGGTGGTGTAGAGCTTCCTGTAGCCGCATGGCGTTCGTACTTAAAT 2000

ATGGAACTAACCATTCCAATTTTCGCTACGAATTCCGACTGCGAGCTTATTGTTAAGGCAATGCAAGGTCTCCTAAAAGATGGAAACCCGATTCCCTCAG 2100

CAATCGCAGCAAACTCCGGCATCTACTAATAGACGCCGGCCA**gcggccgc**ataatgcttaagtcgaacagaaagtaatcgtattgtacacggccgcataa 2200

tcgaaattaatacgactcactataggggaattgtgagcggataacaattccccatcttagtatattagttaagtataagaaggagatata**catatgCGCT** 2300

**TCCGTCAAACCCCTAAACCGGATGATAGACCTCACCTCCCCGCCCAATACTGAAATCTCATTAATACGCATACCCCCACTATACACACGCAATCACCACA** 2400

**TTAGCACAATGAATAATCATCGTACGGGAGAAAACATTCTAAACCC***ACATGAGGATCACCCATGT***cctagg**ctgctgccaccgctgagcaataactagca 2500

taaccccttggggcctctaaacgggtcttgaggggttttttgctgaaacctcaggcatttgagaagcacacggtcacactgcttccggtagtcaataaac 2600

cggtaaaccagcaatagacataagcggctatttaacgaccctgccctgaaccgacgaccgggtcgaatttgctttcgaatttctgccattcatccgctta 2700

ttatcacttattcaggcgtagcaccaggcgtttaagggcaccaataactgccttaaaaaaattacgccccgccctgccactcatcgcagtactgttgtaa 2800

ttcattaagcattctgccgacatggaagccatcacagacggcatgatgaacctgaatcgccagcggcatcagcaccttgtcgccttgcgtataatatttg 2900

cccatagtgaaaacgggggcgaagaagttgtccatattggccacgtttaaatcaaaactggtgaaactcacccagggattggctgagacgaaaaacatat 3000

tctcaataaaccctttagggaaataggccaggttttcaccgtaacacgccacatcttgcgaatatatgtgtagaaactgccggaaatcgtcgtggtattc 3100

actccagagcgatgaaaacgtttcagtttgctcatggaaaacggtgtaacaagggtgaacactatcccatatcaccagctcaccgtctttcattgccata 3200

cggaactccggatgagcattcatcaggcgggcaagaatgtgaataaaggccggataaaacttgtgcttatttttctttacggtctttaaaaaggccgtaa 3300

tatccagctgaacggtctggttataggtacattgagcaactgactgaaatgcctcaaaatgttctttacgatgccattgggatatatcaacggtggtata 3400

tccagtgatttttttctccattttagcttccttagctcctgaaaatctcgataactcaaaaaatacgcccggtagtgatcttatttcattatggtgaaag 3500

ttggaacctcttacgtgccgatcaacgtctcattttcgccaaaagttggcccagggcttcccggtatcaacagggacaccaggatttatttattctgcga 3600

agtgatcttccgtcacaggtatttattcggcgcaaagtgcgtcgggtgatgctgccaacttactgatttagtgtatgatggtgtttttgaggtgctccag 3700

tggcttctgtttctatcagctgtccctcctgttcagctactgacggggtggtgcgtaacggcaaaagcaccgccggacatcagcgctagcggagtgtata 3800

ctggcttactatgttggcactgatgagggtgtcagtgaagtgcttcatgtggcaggagaaaaaaggctgcaccggtgcgtcagcagaatatgtgatacag 3900

gatatattccgcttcctcgctcactgactcgctacgctcggtcgttcgactgcggcgagcggaaatggcttacgaacggggcggagatttcctggaagat 4000

gccaggaagatacttaacagggaagtgagagggccgcggcaaagccgtttttccataggctccgcccccctgacaagcatcacgaaatctgacgctcaaa 4100

tcagtggtggcgaaacccgacaggactataaagataccaggcgtttcccctggcggctccctcgtgcgctctcctgttcctgcctttcggtttaccggtg 4200

tcattccgctgttatggccgcgtttgtctcattccacgcctgacactcagttccgggtaggcagttcgctccaagctggactgtatgcacgaaccccccg 4300

ttcagtccgaccgctgcgccttatccggtaactatcgtcttgagtccaacccggaaagacatgcaaaagcaccactggcagcagccactggtaattgatt 4400

tagaggagttagtcttgaagtcatgcgccggttaaggctaaactgaaaggacaagttttggtgactgcgctcctccaagccagttacctcggttcaaaga 4500

gttggtagctcagagaaccttcgaaaaaccgccctgcaaggcggttttttcgttttcagagcaagagattacgcgcagaccaaaacgatctcaagaagat 4600

catcttattaatcagataaaatatttctagatttcagtgcaatttatctcttcaaatgtagcacctgaagtcagccccatacgatataagttgtaattct 4700

catgttagtcatgccccgcgcccaccggaaggagctgactgggttgaaggctctcaagggcatcggtcgagatcccggtgcctaatgagtgagctaactt 4800

acattaattgcgttgcgctcactgcccgctttccagtcgggaaacctgtcgtgccagctgcattaatgaatcggccaacgcgcggggagaggcggtttgc 4900

gtattgggcgccagggtggtttttcttttcaccagtgagacgggcaacagctgattgcccttcaccgcctggccctgagagagttgcagcaagcggtcca 5000

cgctggtttgccccagcaggcgaaaatcctgtttgatggtggttaacggcgggatataacatgagctgtcttcggtatcgtcgtatcccactaccgagat 5100

gtccgcaccaacgcgcagcccggactcggtaatggcgcgcattgcgcccagcgccatctgatcgttggcaaccagcatcgcagtgggaacgatgccctca 5200

ttcagcatttgcatggtttgttgaaaaccggacatggcactccagtcgccttcccgttccgctatcggctgaatttgattgcgagtgagatatttatgcc 5300

agccagccagacgcagacgcgccgagacagaacttaatgggcccgctaacagcgcgatttgctggtgacccaatgcgaccagatgctccacgcccagtcg 5400

cgtaccgtcttcatgggagaaaataatactgttgatgggtgtctggtcagagacatcaagaaataacgccggaacattagtgcaggcagcttccacagca 5500

atggcatcctggtcatccagcggatagttaatgatcagcccactgacgcgttgcgcgagaagattgtgcaccgccgctttacaggcttcgacgccgcttc 5600

gttctaccatcgacaccaccacgctggcacccagttgatcggcgcgagatttaatcgccgcgacaatttgcgacggcgcgtgcagggccagactggaggt 5700

ggcaacgccaatcagcaacgactgtttgcccgccagttgttgtgccacgcggttgggaatgtaattcagctccgccatcgccgcttccactttttcccgc 5800

gttttcgcagaaacgtggctggcctggttcaccacgcgggaaacggtctgataagagacaccggcatactctgcgacatcgtataacgttactggtttca 5900

cattcaccaccctgaattgactctcttccgggcgctatcatgccataccgcgaaaggttttgcgccattcgatggtgtccgggatctcgacgctctccct 6000

tatgcgactcctgcattaggaaattaatacgactcactata 6041

The original sequence of original pACYCDuet-1 plasmid is shown by black lower cases

The cloning sites of *Nco*I and *Not*I (MCS1 cloning site) are marked in bold red lower cases

The cloning sites of *Nde*I and *Avr*II (MCS2 cloning site) are marked in bold grey lower cases

The cloned sequences are shown in capital letters

MCS1 cloning site

MS2 NcoI and MS2 NotI primer hybridization sites

Maturase (A-proitein) sequence with transversion of A to C (in bold) at position 762

Single-chain coat protein dimer sequence containing His-tag sequence

*Kpn*I restriction sites

MCS2 cloning site

**TMpac – specific control sequence with C-variant of *pac* site (*in italics*)**

# Supplementary Figure 1. Full length *de novo*-synthesized sequence of maturase and single-chain version of the coat protein dimer containing the His-tag. *De novo*-synthesized sequence was used as a PCR template from which the fragment of maturase and single-chain version of the coat protein dimer containing the His-tag was amplified using MS2 NcoI and MS2 NotI primers (primer hybridization sites are underlined) and subsequently cloned to multiple cloning site 1 (MCS1) of the pACYCDuet-1-TM. The sequence in red is the sequence of maturase with marked transversion of A to C (in bold underlined) at position 762 of maturase gene, the sequence in green is the sequence of single-chain coat protein dimer containing His-tag (in blue) surrounded by two *Kpn*I restriction sites (in orange).

**A B**


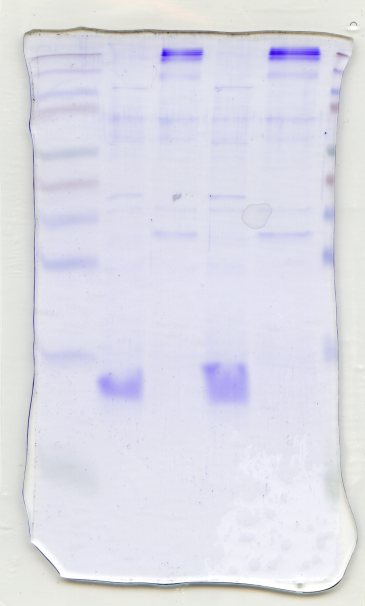

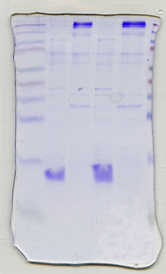


**Supplementary Figure 2.** (A) Full-length sodium dodecyl sulfate-polyacrylamide gel electrophoresis (SDS-PAGE) gel. Two different concentrations of analyzed proteins were tested. (B) Full-length gel with reduced brightness (-20 %) and enhanced contrast (+40 %), figure 5A was cropped form this modified full-length gel.

**A B**


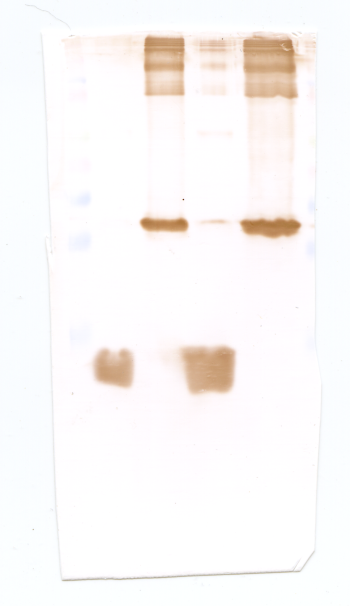

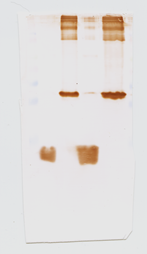


**Supplementary Figure 3.** (A) Full-length western blot analysis results. Two different concentrations of analyzed proteins were tested. (B) Full-length blot with reduced brightness (-20 %) and enhanced contrast (+40 %), figure 5B was cropped form this modified full-length blot.

**A B**


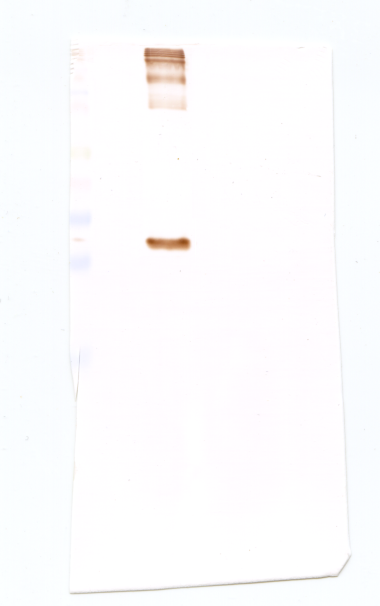

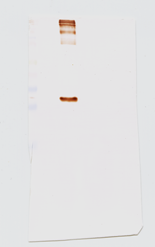


**Supplementary Figure 4.** (A) Full-length western blot analysis results. (B) Full-length blot with reduced brightness (-20 %) and enhanced contrast (+40 %), figure 5C was cropped form this modified full-length blot.


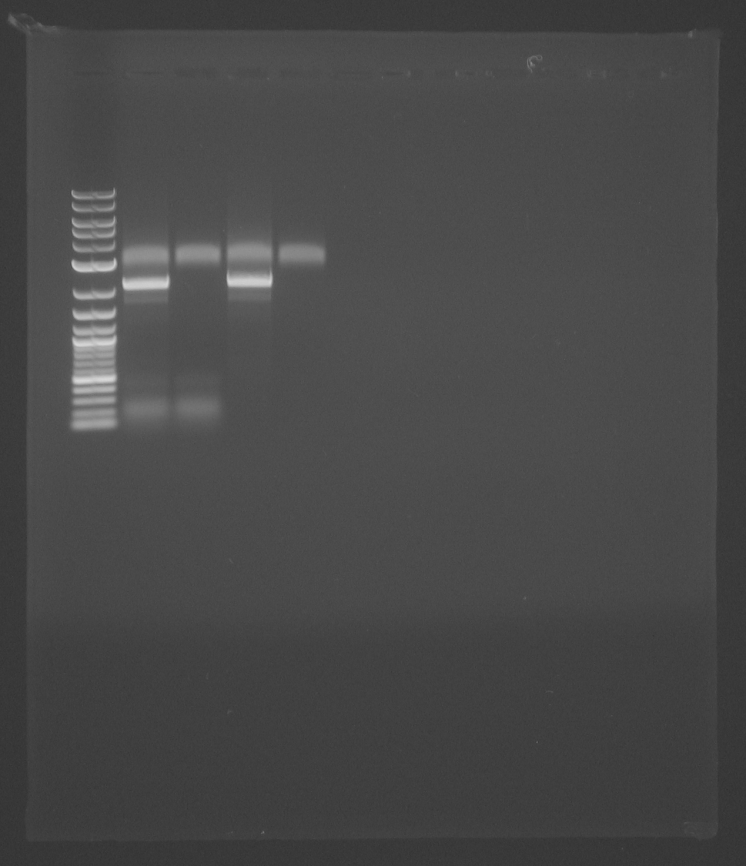


**Supplementary Figure 5.** Full-length agarose gel electrophoresis of His-tagged MS2 phage-like particles (His-tagged MS2 PLP) particles stability testing against nucleases, figure 6 was cropped from this full-length gel.


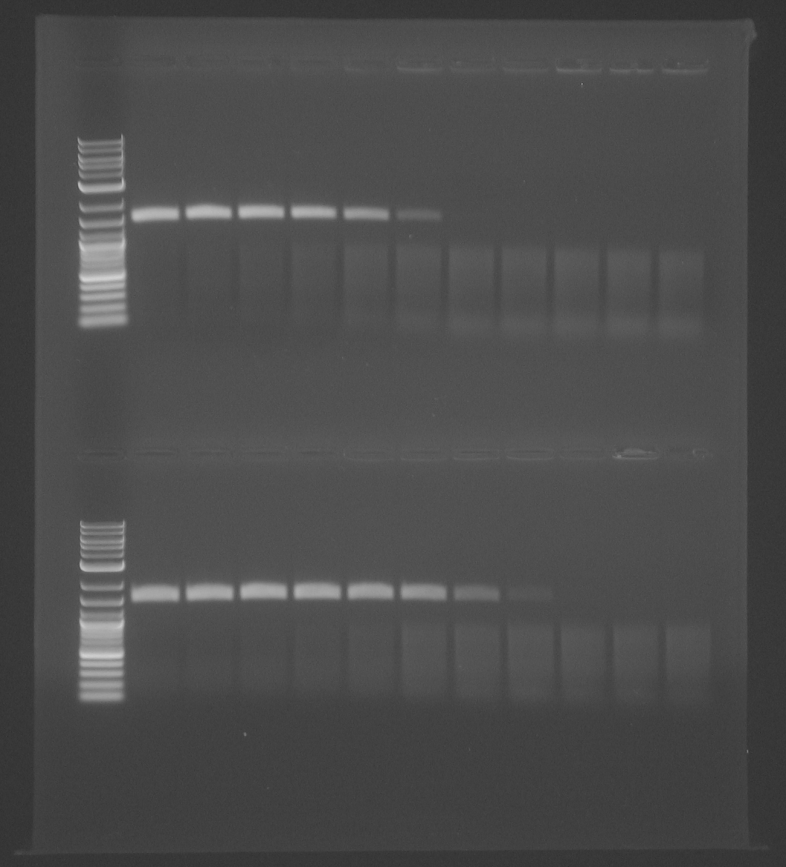


**Supplementary Figure 6.** Full-length agarose gel electrophoresis of MS2 phage-like particles (MS2 PLP) temperature stability testing, figure 7A was cropped from this full-length gel.


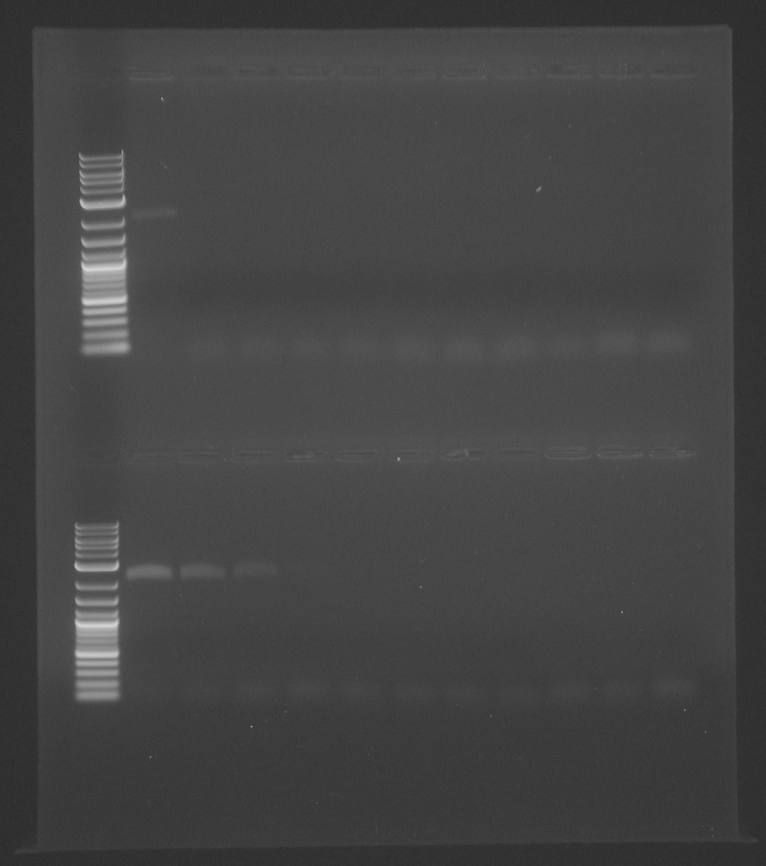


**Supplementary Figure 7.** Full-length agarose gel electrophoresis of His-tagged MS2 phage-like particles (His-tagged MS2 PLP) temperature stability testing, figure 7B was cropped from this full-length gel.
